# Value of TERT promoter mutations for early outcomes in papillary thyroid cancer

**DOI:** 10.1530/ERC-25-0424

**Published:** 2026-08-21

**Authors:** Min Jhi Kim, Daham Kim, Hye Sun Lee, Se Hee Park, Eun Jin Kim, Jin Kyong Kim, Sang-Wook Kang, Jandee Lee, Jong Ju Jeong, Woong Youn Chung, Kee-Hyun Nam

**Affiliations:** ^1^ Department of Surgery, CHA Ilsan Medical Center, CHA University School of Medicine, Goyang-si, Gyeonggi-do, South Korea; ^2^ Department of Internal Medicine, Institute of Endocrine Research, Yonsei University College of Medicine, Seoul, South Korea; ^3^ Biostatistics Collaboration Unit, Yonsei University College of Medicine, Seoul, South Korea; ^4^ Department of Surgery, Yonsei University College of Medicine, Seoul, South Korea

**Keywords:** papillary thyroid cancer, TERT promoter mutation, event-free survival, prognostic factor, propensity score matching

## Abstract

Telomerase reverse transcriptase (TERT) promoter mutations are associated with aggressive clinicopathological features of papillary thyroid cancer (PTC). However, their independent prognostic value remains unclear. This study aimed to evaluate the prognostic significance of TERT promoter mutations (TPMs) in predicting early treatment outcomes and event-free survival (EFS) in patients with PTC. We retrospectively analyzed a prospective cohort; patients underwent surgery at a single tertiary referral center between 2019 and 2022. Patients underwent thyroidectomy, selective postoperative radioactive iodine ablation, and levothyroxine suppression therapy. Propensity score matching (PSM, 1:1) was applied to adjust for baseline clinicopathological differences. Of 10,642 patients with available molecular data, 115 (1.1%) harbored TPMs. After PSM, 90 matched pairs of patients with TERT-wild-type and TERT-mutant tumors were analyzed. Early treatment responses at 1 and 2 years post-treatment and EFS were evaluated. Early treatment responses did not differ significantly between groups at 1 year (*P* = 0.212) and 2 years (*P* = 0.571). However, patients with TERT-mutant tumors had fewer excellent responses and higher rates of structural incomplete response. During follow-up, the TERT-mutant group experienced more recurrences and one disease-specific death. Cumulative EFS was significantly poorer in the TERT-mutant group than in the TERT-wild-type group (*P* = 0.022). Despite their low prevalence, TPMs were independently associated with adverse oncological outcomes, including higher rates of recurrence and mortality. TPMs may serve as valuable prognostic markers for early risk stratification in PTC.

## Introduction

Cellular immortality is a defining hallmark of cancer and primarily achieved by preventing telomere shortening. Telomere erosion during cell division leads to genomic instability and senescence, whereas telomerase maintains telomere length by adding TTAGGG repeats. The telomerase reverse transcriptase (TERT) gene encodes the catalytic subunit of telomerase, and the promoter mutations can reactivate its expression (1, 2). The two most common mutations, c.–124C>T (C228T) and c.–146C>T (C250T), generate novel mitogen-activated protein kinase (MAPK)-dependent E26 transformation-specific transcription factor-binding motifs, resulting in TERT overexpression and cell immortalization (3, 4, 5).

TERT promoter mutations (TPMs) have been identified in various types of cancers, including thyroid cancer (6, 7, 8). The incidence of thyroid cancer ranges from 5.7 to 17.0%, with higher prevalence in aggressive subtypes, such as poorly differentiated and anaplastic thyroid carcinoma, than in differentiated thyroid cancer (DTC) (3, 5, 9). Furthermore, multiple studies have demonstrated the positive association of TPMs in aggressive thyroid cancer with poor clinicopathological characteristics (3, 5, 10, 11).

Recently, thyroid cancer incidence has increased globally, particularly in South Korea (12, 13). Although papillary thyroid cancer (PTC), which represents over 95% of cases, generally has an excellent prognosis, approximately 10–20% exhibit aggressive features with poor outcomes (13, 14, 15, 16, 17). Accordingly, the incidence, clinicopathological correlates, and prognostic implications of TPMs in PTC have been widely investigated.

TPMs in PTC are often evaluated alongside the BRAF V600E mutation, the most common and well-established marker of adverse outcomes (18, 19, 20). Evidence suggests that their co-occurrence exerts synergistic effects, driving more aggressive behavior and a worst prognosis (21, 22, 23, 24). However, the relatively low frequency of TPMs and the high prevalence of BRAF V600E mutations in PTC complicate the determination of the independent prognostic impact of TERT alterations (25, 26, 27, 28).

Our previous study on TERT promoter and BRAF V600E mutations in PTCs demonstrated consistent findings: TPMs were rare but correlated with aggressive clinicopathological characteristics (29). Building on this, we subsequently aimed to investigate the prognostic significance of TPMs in PTC. Specifically, we applied propensity score matching (PSM) to balance baseline clinicopathological factors and then evaluated the impact of TPMs on initial treatment response and early oncological outcomes in matched cohorts.

## Materials and methods

### Patients

A prospectively designed database was reviewed to identify 11,936 consecutive patients with PTC who underwent surgery at the Department of Surgery, Severance Hospital, Yonsei University College of Medicine, between January 2019 and December 2022. Surgical management and adjuvant radioactive iodine (RAI) therapy were determined based on clinical indications. Preoperative evaluations and surgical procedures followed standardized institutional protocols described in detail elsewhere (30).

**Figure 1:**
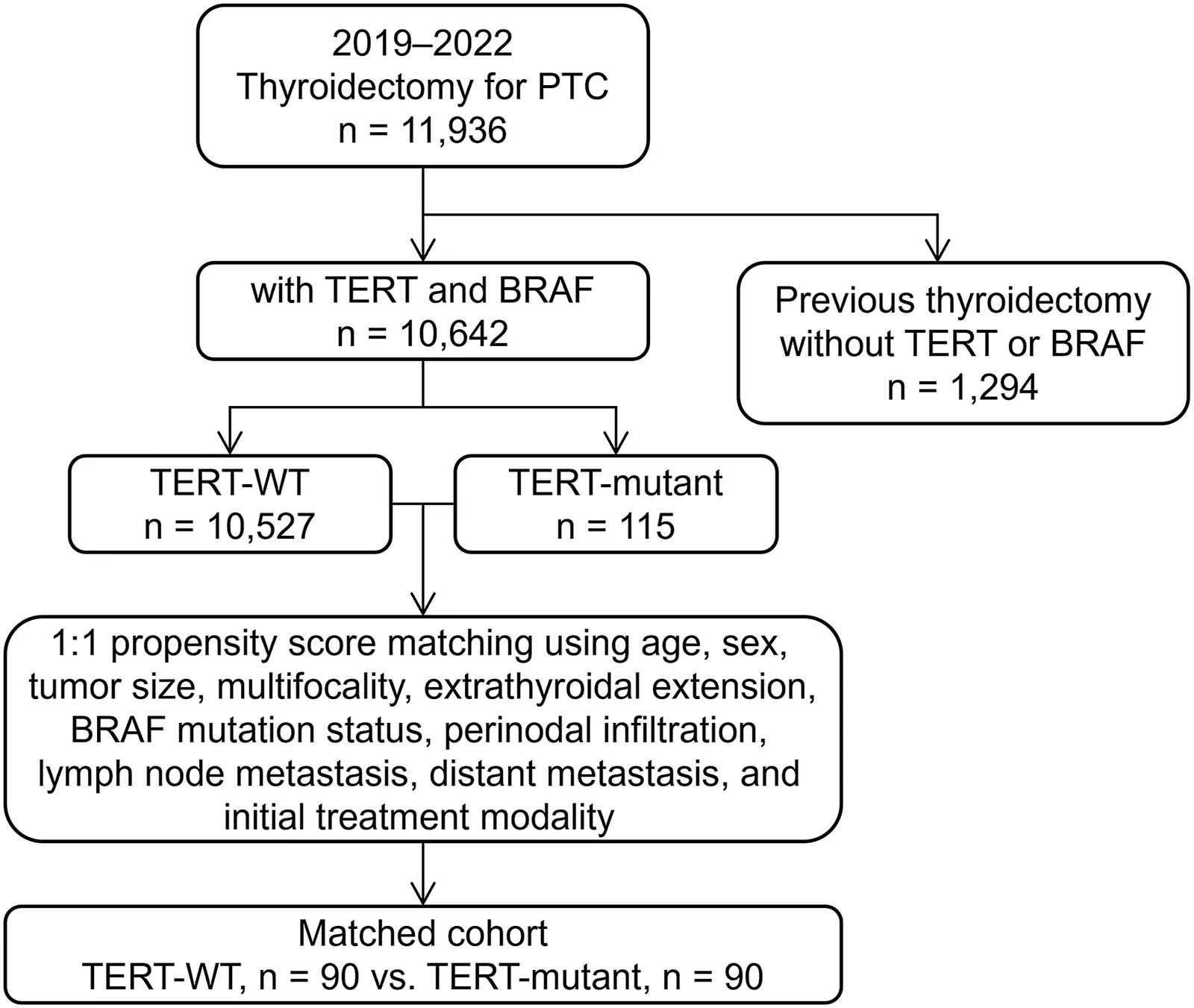
Initial patient selection process using 1:1 propensity score matching. PTC, papillary thyroid cancer; TERT, telomerase reverse transcriptase; WT, wild type. A full color version of this figure is available at https://doi.org/10.1530/ERC-25-0424.

Patients were excluded if they declined mutational analysis, had incomplete clinical, radiological, or pathological data, or had a prior history of cancer or thyroid surgery. After applying these criteria, 10,642 eligible patients were included in the analysis (Fig. 1). Clinical and demographic characteristics were systematically assessed.

### Histopathological and molecular analyses

Histopathological diagnoses were confirmed by experienced thyroid pathologists according to the 2022 World Health Organization classification of thyroid tumors (31). Tumor–node–metastasis staging was assigned based on the 8th edition of the American Joint Committee on Cancer staging system (32, 33). Papillary microcarcinoma (PMC) was defined as a tumor measuring ≤1 cm in maximum diameter.

Mutational analyses for the TERT promoter and BRAF V600E were performed as previously reported (29). Genomic DNA was extracted from formalin-fixed, paraffin-embedded tumor specimens. The main tumor was analyzed in multifocal lesions, and both lesions were tested for bilateral tumors.

TERT promoter (C228T and C250T) and BRAF V600E mutations were assessed using pyrosequencing. Polymerase chain reaction amplification was followed by pyrosequencing reaction on a PyroMark Q24 instrument (Qiagen, Germany). Data were analyzed with PyroMark Q24 software to differentiate mutant from wild-type (WT) alleles (34). Primer sequences have been reported previously (29).

### Outcome assessment

All patients received levothyroxine postoperatively for thyroid-stimulating hormone (TSH) suppression or hormone replacement, as indicated. Adjuvant RAI ablation was considered in patients who underwent total thyroidectomy (TT), according to the American Thyroid Association (ATA) guidelines (32, 33, 35, 36).

TSH-stimulated serum thyroglobulin (Tg) levels were measured before RAI administration to evaluate surgical completeness (37). Post-treatment surveillance included measurement of TSH-suppressed Tg and anti-Tg antibody levels at 3 and 6 months and annually thereafter in patients who had undergone TT. Cervical ultrasonography was performed every 6–12 months to monitor recurrence. Additional imaging, such as neck computed tomography (CT) or positron emission tomography-CT, was performed when recurrence was suspected. Regional lymph node (LN) recurrence was confirmed through fine-needle aspiration biopsy.

Treatment response was evaluated annually until the last follow-up using the ATA response-to-therapy classification and dynamic risk stratification systems (35, 38). Treatment responses were categorized as excellent response (ER), biochemical incomplete response (BIR), structural incomplete response (SIR), and indeterminate response (IR).

Disease-specific mortality was defined as death directly attributable to PTC progression. For the purpose of event-free survival (EFS) analysis, an event was defined as structural recurrence, distant metastasis, or disease-specific death. EFS was measured from the date of initial treatment to certain events or the date of the last follow-up.

### Statistical analysis

Categorical variables are presented as counts and percentages and continuous variables as mean ± standard deviation. Between-group categorical variables were compared using the chi-squared (*χ*^2^) or Fisher’s exact test. Continuous variables were compared using Student’s *t*-test for two groups.

Because of the low prevalence of TPMs in our cohort (1.1%), 1:1 PSM was performed to minimize confounding. Ten clinicopathological variables were included in the matching model: age, sex, tumor size, tumor multifocality, extrathyroidal invasion of the tumor, BRAF V600E mutation, LN metastasis, perinodal infiltration, distant metastasis, and initial treatment modalities (33, 35, 39, 40). A caliper width 0.2 of the pooled standard deviation of the logit of the propensity score was used. Adequate covariate balance was confirmed by absolute standardized differences <0.20 (41). After PSM, 90 patients per group were analyzed for early treatment response and EFS were compared.

EFS was determined using the Kaplan–Meier method and compared using the log-rank test. The magnitude of the association between TPMs and EFS was quantified using restricted mean survival time with 95% confidence intervals. Statistical significance was set at *P* < 0.05. PSM was performed using SAS (version 9.4; SAS Institute, USA) and R (version 4.3.2; R Foundation for Statistical Computing, Austria). All other analyses were performed using SPSS Statistics for Windows (version 25.0; IBM Corp., USA).

### Ethics statement

The study was conducted in accordance with the principles of the Declaration of Helsinki (1964, and subsequent amendments) and was approved by the Institutional Review Board of the Severance Hospital (4-2025-0882). Written informed consent was obtained from all patients for perioperative procedures and mutational analyses of PTCs.

## Results

### TPMs and characteristics of the overall and propensity score-matched patient groups

Among the 10,642 patients analyzed, TPMs were identified in 1.1%, while 98.9% were TERT-WT (Table 1). Compared with the TERT-WT group, patients with TPMs were significantly older (*P* < 0.0001) and more frequently male (*P* < 0.0001).

**Table 1:** Clinicopathological features of patients with TERT-wild-type and TERT-mutant PTC before and after propensity score matching.

Variables	Before matching				After matching			
	TERT-WT	TERT-mutant	*P*-value	Absolute standardized difference	TERT-WT	TERT-mutant	*P*-value	Absolute standardized difference
Number of cases (%)	10,527 (98.9)	115 (1.1)			90 (100.0)	90 (100.0)		
Age (year)	42.9 ± 12.1	61.9 ± 11.8	<0.0001	1.59	57.6 ± 14.5	58.7 ± 11.0	0.50	0.08
Sex, male	2,449 (23.3)	51 (44.4)	<0.0001	0.46	40 (44.4)	39 (43.3)	0.87	0.02
Tumor size (cm)	1.0 ± 0.7	2.0 ± 1.3	<0.0001	1.01	1.9 ± 1.451	1.7 ± 1.0	0.37	0.13
Multifocality	3,590 (34.1)	57 (49.6)	0.0005	0.32	47 (52.2)	42 (46.7)	0.37	0.11
Extrathyroidal extension	915 (8.7)	58 (50.4)	<0.0001	1.03	38 (42.2)	37 (41.1)	0.86	0.02
BRAF V600E mutation	7,632 (72.5)	94 (81.7)	0.0271	0.22	76 (84.4)	74 (82.2)	0.68	0.06
Perinodal infiltration	878 (8.3)	45 (39.1)	<0.0001	0.78	32 (35.6)	29 (32.2)	0.61	0.07
LN metastasis	4,772 (45.3)	66 (57.4)	0.0098	0.24	52 (57.8)	49 (54.4)	0.63	0.07
Distant metastasis	25 (0.2)	10 (8.7)	<0.0001	0.42	1 (1.1)	2 (2.2)	0.56	0.09
Initial treatment modality			<0.0001				0.41	
TT + RAI	2,004 (19.0)	68 (59.1)		0.90	49 (54.4)	48 (53.3)		0.02
TT only	1,016 (9.7)	13 (11.3)		0.05	12 (13.3)	8 (8.9)		0.14
Lobectomy	7,507 (71.3)	34 (29.6)		0.92	29 (32.2)	34 (37.8)		0.12

Values are expressed as mean ± standard deviation or number (%).

TERT, telomerase reverse transcriptase; WT, wild type; PTC, papillary thyroid cancer; LN, lymph node; TT, total thyroidectomy; RAI, radioactive iodine.

The TERT-mutant group had significantly larger tumors (*P* < 0.0001), more frequent multifocality (*P* = 0.0005) and extrathyroidal extension (*P* < 0.0001), and a higher prevalence of BRAF V600E mutations (*P* = 0.0271). LN metastases (*P* = 0.0098) with perinodal infiltration (*P* < 0.0001) were frequently observed in the TERT-mutant group. Distant metastases were also highly prevalent (*P* < 0.0001).

Reflecting these adverse features, patients in the TERT-mutant group more often underwent TT with or without RAI, whereas those in the TERT-WT group more frequently had lobectomy (*P* < 0.0001).

To reduce cofounding, PSM was applied, producing 90 matched pairs (Fig. 1). After matching, no significant differences were observed between groups in the ten covariates used for matching (Table 1).

### TPMs and 1-year treatment response

The relationship between TPMs and the treatment response 1 year after initial therapy was evaluated in the matched cohort (Table 2). In the TERT-WT group, outcomes were 75.6% ER, 14.4% IR, 8.9% BIR, and 1.1% SIR. In the TERT-mutant group, rates were 68.9% ER, 16.7% IR, 10.0% BIR, and 4.4% SIR. The difference was not significant (*P* = 0.212).

**Table 2:** One-year treatment response according to the TERT mutation status and RAI ablation therapy.

Treatment response	TERT-WT All (*n* = 90)	TERT-mutant All (*n* = 90)	TERT-WT RAI (*n* = 49)	TERT-mutant RAI (*n* = 48)	TERT-WT No RAI (*n* = 41)	TERT-mutant No RAI (*n* = 42)	*P*-value All	*P*-value RAI	*P*-value No RAI
Excellent	68 (75.6)	62 (68.9)	34 (69.4)	29 (60.4)	34 (82.9)	33 (78.6)	0.212	0.274	0.484
Indeterminate	13 (14.4)	15 (16.7)	8 (16.3)	10 (20.8)	5 (12.2)	5 (11.9)			
Biochemical incomplete	8 (8.9)	9 (10.0)	6 (12.3)	5 (10.4)	2 (4.9)	4 (9.5)			
Structural incomplete	1 (1.1)	4 (4.4)	1 (2.0)	4 (8.4)	0 (0.0)	0 (0.0)			

Values are expressed as numbers (%). *P*-values were calculated using the chi-squared (*χ*^2^) or Fisher’s exact test.

TERT, telomerase reverse transcriptase; WT, wild type; RAI, radioactive iodine.

No significant differences in the treatment responses were observed according to TPMs among the patients who received TT with RAI therapy (TERT-WT: 69.4% ER, 16.3% IR, 12.3% BIR, and 2.0% SIR vs TERT-mutant: 60.4% ER, 20.8% IR, 10.4% BIR, and 8.4% SIR; *P* = 0.274). Treatment responses in those who underwent surgery alone were similar in the two groups (TERT-WT: 82.9% ER, 12.2% IR, 4.9% BIR, and 0% SIR vs TERT-mutant: 78.6% ER, 11.9% IR, 9.5% BIR, and 0% SIR; *P* = 0.484).

Further subgroup analyses were performed in patients who received TT followed by RAI (Table 3). In patients with the BRAF V600E mutation, treatment responses showed no considerable difference between the TERT-WT (70.5% ER, 15.9% IR, 11.4% BIR, and 2.2% SIR) and TERT-mutant (62.5% ER, 20.0% IR, 10.0% BIR, and 7.5% SIR) groups (*P* = 0.370). Treatment responses in patients without distant metastases were not significantly different according to TPM (TERT-WT: 70.8% ER, 16.7% IR, 12.5% BIR, and 0% SIR vs TERT-mutant: 63.0% ER, 21.7% IR, 10.9% BIR, and 4.4% SIR; *P* = 0.360).

**Table 3:** One-year treatment response according to the BRAF V600E mutation status and the absence of distant metastasis in patients who underwent RAI ablation.

Treatment response	TERT-WT RAI BRAF V600E (*n* = 44)	TERT-mutant RAI BRAF V600E (*n* = 40)	TERT-WT RAI M0 stage (*n* = 48)	TERT-mutant RAI M0 stage (*n* = 46)	*P*-value RAI BRAF V600E	*P*-value RAI M0 stage
Excellent	31 (70.5)	25 (62.5)	34 (70.8)	29 (63.0)	0.370	0.360
Indeterminate	7 (15.9)	8 (20.0)	8 (16.7)	10 (21.7)		
Biochemical incomplete	5 (11.4)	4 (10.0)	6 (12.5)	5 (10.9)		
Structural incomplete	1 (2.2)	3 (7.5)	0 (0.0)	2 (4.4)		

Values are expressed as numbers (%). *P*-values were calculated using the chi-squared (*χ*^2^) or Fisher’s exact test.

TERT, telomerase reverse transcriptase; WT, wild type; RAI, radioactive iodine.

### TPMs and treatment response at 2 years after initial treatment

Treatment responses at 2 years post-treatment were investigated in the matched cohorts (Table 4). No significant difference in the overall treatment response was observed between the groups (TERT-WT: 79.2% ER, 9.8% IR, 9.8% BIR, and 1.2% SIR vs TERT-mutant: 73.8% ER, 15.5% IR, 8.3% BIR, and 2.4% SIR; *P* = 0.571).

**Table 4:** Two-year treatment response according to the TERT mutation status and RAI ablation therapy.

Treatment response	TERT-WT All (*n* = 82)	TERT-mutant All (*n* = 84)	TERT-WT RAI (*n* = 47)	TERT-mutant RAI (*n* = 45)	TERT-WT No RAI (*n* = 35)	TERT-mutant No RAI (*n* = 39)	*P*-value All	*P*-value RAI	*P*-value No RAI
Excellent	65 (79.2)	62 (73.8)	35 (74.5)	28 (62.2)	30 (85.7)	34 (87.1)	0.571	0.322	0.679
Indeterminate	8 (9.8)	13 (15.5)	5 (10.6)	9 (20.0)	3 (8.6)	4 (10.3)			
Biochemical incomplete	8 (9.8)	7 (8.3)	6 (12.8)	6 (13.3)	2 (5.7)	1 (2.6)			
Structural incomplete	1 (1.2)	2 (2.4)	1 (2.1)	2 (4.5)	0 (0.0)	0 (0.0)			

Values are expressed as numbers (%). *P*-values were calculated using the chi-squared (*χ*^2^) or Fisher’s exact test.

TERT, telomerase reverse transcriptase; WT, wild type; RAI, radioactive iodine.

Among patients who underwent TT with RAI, treatment responses were also not significantly different between the two groups (TERT-WT: 74.5% ER, 10.6% IR, 12.8% BIR, and 2.1% SIR vs TERT-mutant: 62.2% ER, 20.0% IR, 13.3% BIR, and 4.5% SIR; *P* = 0.322). In those treated with surgery alone, responses remained comparable (TERT-WT: 85.7% ER, 8.6% IR, 5.7% BIR, and 0% SIR vs TERT-mutant: 87.1% ER, 10.3% IR, 2.6% BIR, and 0% SIR; *P* = 0.679).

In patients who underwent TT and RAI, further analyses revealed no significant difference in the treatment responses according to TPMs among patients with BRAF V600E mutation (TERT-WT: 73.8% ER, 11.9% IR, 11.9% BIR, and 2.4% SIR vs TERT-mutant: 62.2% ER, 21.6% IR, 13.5% BIR, and 2.7% SIR; *P* = 0.449). The treatment responses in patients without distant metastasis were similar in the TERT-WT (76.0% ER, 10.9% IR, 10.9% BIR, and 2.2% SIR) and TERT-mutant (63.6% ER, 20.5% IR, 13.6% BIR, and 2.3% SIR) groups (*P* = 0.358) (Table 5).

**Table 5:** Two-year treatment response according to the BRAF V600E mutation status and the absence of distant metastasis in patients who underwent RAI ablation.

Treatment response	TERT-WT RAI BRAF V600E (*n* = 42)	TERT-mutant RAI BRAF V600E (*n* = 37)	TERT-WT RAI M0 stage (*n* = 46)	TERT-mutant RAI M0 stage (*n* = 44)	*P*-value RAI BRAF V600E	*P*-value RAI M0 stage
Excellent	31 (73.8)	23 (62.2)	35 (76.0)	28 (63.6)	0.449	0.358
Indeterminate	5 (11.9)	8 (21.6)	5 (10.9)	9 (20.5)		
Biochemical incomplete	5 (11.9)	5 (13.5)	5 (10.9)	6 (13.6)		
Structural incomplete	1 (2.4)	1 (2.7)	1 (2.2)	1 (2.3)		

Values are expressed as numbers (%). *P*-values were calculated using the chi-squared (*χ*^2^) or Fisher’s exact test.

TERT, telomerase reverse transcriptase; WT, wild type; RAI, radioactive iodine.

### TPMs and oncologic outcomes

After a median follow-up of 43.3 months (range, 12–72 months), eight patients were lost to follow-up: three from the TERT-WT group and five from the TERT-mutant group.

Locoregional recurrence occurred in one (1.1%) and five (5.6%) patients in the TERT-WT and TERT-mutant groups, respectively. Among these, two had thyroidectomy bed recurrence and four had LN recurrence. Patients with thyroidectomy bed recurrence underwent reoperation for excision of the recurrent mass with or without RAI therapy, whereas those with LN recurrence underwent neck node dissection followed by high-dose RAI therapy (Table 6).

**Table 6:** Patients with event occurrence during follow-up.

No	TERT-mutant	Initial treatment	Event-free duration (months)	Event site	Treatment modality
1	+	TT + MRND + RAI	54	Op bed recurrence	Excision
2	+	Rt total and Lt partial thyroidectomy	52	LN recurrence	Completion thyroidectomy + CCND + RAI
3	+	TT + MRND + RAI	64	LN recurrence	-
4	+	TT + MRND + osteotomy + RAI	12	Bone metastases	Death
5	+	TT + RAI	44	LN recurrence	MRND + RAI
6	+	TT + RAI	37	LN recurrence	MRND + RAI
7	-	TT + MRND + RAI	24	Op bed recurrence	Excision + RAI

No, number; TERT, telomerase reverse transcriptase; TT, total thyroidectomy; MRND, modified radical neck dissection; RAI, radioactive iodine; Op, operation; LN, lymph node; CCND, central compartment neck dissection.

**Figure 2:**
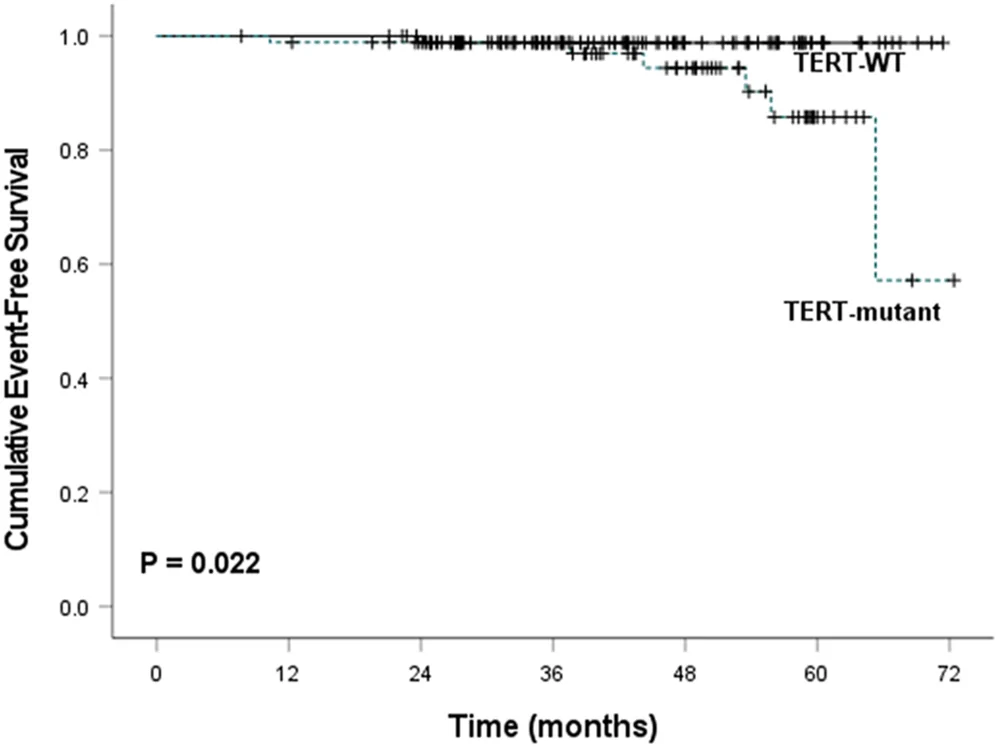
Kaplan–Meier curves of event-free survival in the TERT-WT and TERT-mutant groups after 1:1 propensity score matching. TERT, telomerase reverse transcriptase; WT, wild type. A full color version of this figure is available at https://doi.org/10.1530/ERC-25-0424.

Disease-specific mortality was observed in one patient in the TERT-mutant group who was senile and whose disease progressed to extensive bone metastasis (Table 6). Kaplan–Meier analysis demonstrated significantly worse EFS in the TERT-mutant group compared to the TERT-WT group (log-rank *P* = 0.022) (Fig. 2). The restricted mean event-free survival time was 70.8 months (95% CI: 69.8–71.9) in the TERT-wild-type group and 66.8 months (95% CI: 62.5–71.0) in the TERT-mutant group, corresponding to an absolute difference of approximately 4.1 months.

## Discussion

In PTCs, early treatment response after initial therapy is considered an important surrogate marker of future recurrence and survival (35, 38, 39). Among molecular biomarkers in thyroid cancer, TERT has drawn particular attention due to its direct role in tumorigenesis and aggressiveness (1, 2, 42). TPMs also exhibit synergistic interactions with other oncogenic drivers, such as BRAF V600E, RAS mutations, and RET/PTC rearrangements, all of which activate the RAS-MAPK pathway, a key mechanism for thyroid tumorigenesis (3, 4, 43, 44, 45, 46). Genetic alterations related to TERT re-expression and telomerase activation are generally considered late events in thyroid tumorigenesis. These changes accumulate in advanced thyroid cancers, explaining the higher frequency of TPMs in aggressive types of thyroid cancer (5, 10, 11).

The global rise in PTC incidence over recent decades has spurred continuous efforts to define the prognostic role of TPM in PTC aggressiveness. However, its low prevalence in PTC and the indolent clinical course have limited definitive conclusions, leading to heterogeneous findings across studies (3, 24, 25, 47, 48, 49, 50). The reported prevalence of TPMs in Asian countries has been rarer, ranging from 2.8 to 5.7% (9, 25, 51, 52).

In our previous study of 7,797 patients with PTC, TPM prevalence was 1.1%, one of the lowest rates in the literature (29). The present study, which included most of the prior cohort, confirmed similar findings: TPMs were detected in 1.1% of 10,642 PTC cases and associated with older age, male sex, and adverse clinicopathological features, prompting the use of more aggressive treatment modalities. These findings are consistent with the previous studies (3, 5, 9, 10, 24, 47, 53).

Owing to the extremely rare prevalence of TERT-mutant PTCs, we used 1:1 PSM to evaluate the prognostic utility of TPMs while minimizing confounding. Measured clinicopathological factors were included for matching and well balanced after PSM (33, 35, 39). Given the known synergistic interaction between TPM and BRAF V600E, resulting in a far more aggressive PTC, BRAF V600E was also included in matching to investigate the independent value of TPM (21, 22, 23, 24, 48).

The overall treatment responses at 1 and 2 years after initial treatment were not significantly different according to TPM status. However, numerically lower ER and higher incomplete responses were observed in TERT-mutant cases. The divergence was more pronounced among patients treated with TT and RAI than among those who underwent surgery alone. Specifically, the numerically higher SIR was observed in several patients with TPM, among those who received TT and RAI. However, no patient exhibited SIR among those who underwent surgery alone. Adverse treatment responses were more frequent after aggressive initial therapy, consistent with prior studies showing a positive correlation between TPMs and RAI refractoriness in DTCs, especially in those with distant metastases (54, 55, 56, 57).

Moreover, our previous study showed TPMs were associated with higher TSH-stimulated serum Tg levels before RAI and increased metastatic uptake on post-therapeutic whole-body scans (29). Consistent with these findings, the poorer responses in the TERT-mutant cases treated with TT and RAI in this study suggest that adverse treatment responses are more frequent in advanced thyroid cancers harboring TPMs.

To further clarify these associations, subgroup analyses were conducted among patients receiving RAI, stratified by BRAF V600E mutation status and the presence or absence of distant metastasis (18, 19, 20, 55). No significant differences were observed in initial treatment responses. However, numerical differences were consistently noted, with TERT-mutant cases showing lower rates of ER and higher rates of incomplete response. These consistent trends may add some clues to indicate an independent contribution of TPMs to adverse clinical outcomes in PTC.

At the last follow-up, five locoregional recurrences and one disease-specific death were documented in the TERT-mutant group, compared with one recurrence in the TERT-WT group. The cumulative EFS was significantly poorer in patients with TPMs. These are consistent with the findings of most prior studies and meta-analyses, which have linked TPMs to increased risks of disease persistence/recurrence and disease-specific mortality in PTC (3, 5, 47, 48, 53, 58, 59). While many of these reports are based on long-term follow-up (>5 years), our study, with a relatively shorter median follow-up period of 43.3 months, similarly highlights the clinical relevance of TPMs as markers of poor prognosis in PTC.

Nevertheless, some studies have reported a diminished prognostic impact of TPMs in PTCs (24, 49, 50). Several factors may account for these discrepancies: the predominance of BRAF V600E mutations, the synergistic aggressiveness of BRAF V600E and TERT promoter mutations, the low incidence of TPMs, and the sharply rising incidence of PMCs. Furthermore, different study populations, concurrent gene analyses, statistical methodologies, and follow-up durations may also contribute to these inconsistent findings across the literature.

A key strength of this study is that it represents one of the largest cohorts of patients with PTC reported to date, in which TPMs were evaluated; routine mutational analyses were available for several thousand patients at our institution. However, this study also has some limitations. First, as a single-institution study based on the Korean population, the findings may not be generalizable to other ethnicities or practice settings. The well-established health screening services in Korea likely contributed to the higher detection of early-stage PMCs, which may explain the rare prevalence of TPMs. Second, the rarity of TPMs allowed for further evaluation after PSM; however, regression analyses of potential prognostic factors could not be performed reliably. Third, although the study was initially designed as a prospective cohort, data were retrospectively reviewed, introducing the possibility of selection and information bias. Fourth, the median follow-up period was 43.3 months. While most PTC recurrences occur within 5 years of initial treatment, longer follow-up is necessary to fully capture late events (60). Finally, these limitations have hindered further evaluation on the impact of coexisting BRAF V600E and TERT promoter mutation on the oncological outcomes of PTC.

In this largest single-institution cohort of patients with PTC in Korea, early treatment responses did not differ significantly according to TPM status. However, significantly increased adverse oncological events such as cancer recurrence or mortality, along with numerically higher rates of incomplete responses in TERT-mutant PTCs, may support the independent prognostic potential of TPMs in PTC. The meaningful results, despite the low prevalence of TPM and the relatively short follow-up, represent notable strengths of this study. Recognition of TPMs as clinically meaningful biomarkers could inform more tailored treatment strategies and enhance early risk stratification. Future multicenter studies with larger cohorts and extended follow-up will be critical to validate these observations.

## Declaration of interest

The authors declare that there is no conflict of interest that could be perceived as prejudicing the impartiality of the work reported.

## Funding

This research did not receive any specific grant from any funding agency in the public, commercial, or not-for-profit sector.

## Author contribution statement

DK and MJK conceived and designed the study. DK acquired the data. DK and MJK analyzed and/or interpreted the data. MJK drafted the original manuscript. MJK, DK, and KHN critically revised the manuscript for important intellectual content. MJK, DK, JKK, SWK, JL, JJJ, WYC, and KHN reviewed and approved the final version for publication.

## Data availability

Some or all data generated or analyzed during this study are included in this published article or in the data repositories listed in references.
